# The Developmental Competence of Oocytes Retrieved from The Leading Follicle in Controlled Ovarian Stimulated Cycles

**Published:** 2013-03-03

**Authors:** Daniela Paes de Almeida Ferreira Braga, Tatiana Carvalho de Souza Bonetti, Ismael Dale Cotrim Guerreiro da Silva, Amanda Souza Setti, Assumpto Jr Iaconelli, Edson Jr Borges

**Affiliations:** 1 Fertility - Assisted Fertilization Center, Sao Paulo, Brazil; 2 Sapientiae Institute, Educational and Research Center in Assisted Reproduction, Sao Paulo, Brazil; 3 Molecular Gynecology Laboratory, Departament of Gynecology, University Federal of São Paulo, Sao Paulo - SP, Brazil

**Keywords:** Oocyte Retrieval, Ovarian Stimulation, Vacuolization, Intracytoplasmic Sperm Injection

## Abstract

**Background:**

This study compares the developmental capacity of gametes retrieved from the largest follicle with small follicles of a cohort in controlled ovarian stimulated cycles.

**Materials and Methods:**

This prospective study performed in a private assisted fertilization center included 1016 follicles collected from 96 patients who underwent intra cytoplasmic sperm injection (ICSI). After follicular aspiration, oocytes were assigned to two groups according to the diameter of the derived follicle. The large follicle group (n=96) comprised oocytes derived from the leading follicle of the cohort and the small follicle group (n=920) consisted oocytes derived from the smaller follicles of the cohort. The fertilization and percentage of topquality embryos were compared between groups by Chi-square or Fisher’s exact test, where appropriate. The effect of the follicular diameter on oocyte dimorphism was assessed by binary logistic regression.

**Results:**

A significantly higher percentage of oocytes derived from the leading follicle were in the metaphase II (MII) stage (100 vs. 70.0%, p<0.001). However we observed no significant differences regarding the percentage of degenerated oocytes between the large (6.25%) and small follicle (5.0%) groups (p=0.550). Regression analysis demonstrated a nearly two-fold increase in the incidence of vacuoles in oocytes derived from the largest follicle of the cohort (OR: 1.81, p=0.046). The fertilization rate (50.0 vs. 38.8%, p=0.038) and the percentage of top quality embryos (84.7 vs. 76.4%, p=0.040) were significantly higher for oocytes derived from the largest follicle. However, the percentage of abnormal fertilized oocytes was equally distributed between the large follicle (15.0%) and small follicle (12. 8%) groups (p=0.550).

**Conclusion:**

Our data suggest that intrafollicular mechanisms within the larger follicle of the cohort may allow for these follicles to amplify the responsiveness to exogenous gonadotropin, which leads to the formation of more competent oocytes with higher fertilization and developmental capacities.

## Introduction

Ovarian folliculogenesis is a complex process involving interactions between the classical hypothalamus- pituitary-ovarian axis and other intra- and extra-ovarian factors (1). Up to a certain point, follicular growth and development occur readily in the presence of normal basal concentrations of gonadotropin, metabolic hormones and growth factors, but the follicles eventually reach the end of their normal life span under those basal conditions. At that time, only the follicle exposed to specific additional signals (dominant follicle) will continue to grow until ovulation while the remainder (subordinate follicles) become atretic and regress (2-4). This process, known as follicle selection, involves a reduction in systemic follicle stimulation hormone (FSH) concentrations below the concentration required by the smaller follicles (4). This depressed FSH concentration is maintained by a negative feedback loop of protein produced by the dominant follicle such as inhibin (5) and estradiol (4).

The ability of the dominant follicle to continue growth under decreased FSH concentrations, while the subordinate follicles regress, suggests that responsiveness to FSH or FSH dependence may be altered during follicular development (4). Indeed, the largest follicle acquires luteinizing hormone (LH) receptors or gene expression for LH receptors between two and four days after wave emergence (6, 7). Follicle selection involves a transient elevation in LH, which is required for the production of estradiol and free insulin-like growth factor-I (IGF-I). IGF-I synergizes with FSH to stimulate granulosa cell proliferation and steroidogenesis (8). The smaller follicles have not reached a similar developmental stage and, because of their dependency on FSH, they become susceptible to low FSH concentrations (9, 10).

During controlled ovarian stimulation (COS), women are usually treated with an agonist or antagonist of gonadotropin-releasing hormone (GnRH) to block the action of the pituitary, and their ovaries are stimulated with gonadotropins to induce the development and final maturation of multiple follicles (11). Therefore, the increase in circulating levels of gonadotropins will override the selection of a single dominant follicle and stimulate the development of multiple antral follicles whose enclosed oocytes have the potential for fertilization and further development (12).

In monovular animal species, several intrafollicular events occur before the beginning of diameter deviation between the largest follicle and the second largest follicle of the cohort. Therefore, although mature oocytes may be retrieved from multiple follicles after COS, it is still a matter of debate whether oocytes retrieved from small follicles, which have escaped from atresia under supraphysiologic doses of gonadotropins, present the same developmental competence as oocytes derived from larger follicles (12).

The present study compared the developmental capacity of gametes retrieved from the largest follicle and the other small follicles of the cohort in COS intracytoplasmic sperm injection (ICSI) cycles.

## Materials and Methods

### Experimental design

This prospective study included a total of 1016 follicles collected from 96 patients who underwent ICSI cycles in the Fertility-Assisted Fertilization Center, Brazil between January 2007 and December 2008.

After follicular aspiration, we assigned the oocytes to two groups according to the diameter of the derived follicle: i. oocytes derived from the leading follicle of the cohort (large follicle, n=96) and ii. oocytes derived from the smaller follicles of the cohort (small follicle, n=920). The fertilization and percentage of top-quality embryos were compared between groups. We assessed the effect of follicular diameter on oocyte dimorphism.

The patients’ ages ranged from 22 to 43 years old (median ± SEM: 33.4 ± 0.42). All patients presented with the following inclusion criteria: the presence of both ovaries, a regular menstrual cycle, BMI lower than 35 kg/m^2^, no ongoing infectious diseases, no uterine pathology, basal FSH <14 IU/ml and basal E2 <70 pg/ml. All ejaculated semen used for ICSI presented motile sperm concentrations above 5 × 10^6^ sperm/ml.

Infertility was defined as unexplained infertility (28/96: 29.1%), male infertility (27/96: 28.1%), male- and female-associated factors (12/96: 12.5%), endometriosis (10/96: 10.4%), ovarian factors (11/96: 11.4%) and tubal obstructions (8/96: 8.3%).

### Controlled ovarian stimulation

COS was achieved by pituitary blockage using a GnRH antagonist (Cetrotide, Serono, Geneva, Switzerland) and by ovarian stimulation with recombinant- FSH (Gonal-F®, Serono, Geneva, Switzerland). The patients began daily recombinant-FSH treatment (225 IU) from the third day of their menstrual cycles. The first ultrasound control and the E2 plasma dosage tests were performed at the seventh cycle day. Depending on the response of each patient that was determined by ultrasound monitoring of the follicle size, we adjusted the dose of recombinant-FSH. GnRH antagonist was administered when the dominant follicle was 14 mm in diameter. When at least three follicles reached 18 mm in diameter and serum estradiol level reached >600 pg/mL, recombinant human chorionic gonadotropin (r-hCG, Ovidrel™, Serono, Geneva, Switzerland) was administered to trigger final follicular maturation. Oocytes were collected 35 hours after hCG administration by transvaginal ultrasound ovum pick-up.

The leading follicles (largest follicle of both ovaries) were the first to be aspirated; smaller follicles were subsequently aspirated in different tubes.

### Preparation of oocytes

Briefly, after retrieval, oocytes were incubated in culture medium (G-MOPS™-V1, Vitrolife, Kungsbacka, Sweden) covered with mineral oil (Ovoil™, Vitrolife, Kungsbacka, Sweden) at 37ºC and 6% CO_2_ for 5 hours, according to the previously established protocol (13). The oocyte retrieved from the largest follicle was cultured in a different drop from the other oocytes. Cumulus cells were removed with a 30 second exposure to Hepes-buffered medium that contained 80 IU/mL hyaluronidase (Irvine Scientific, Santa Ana, USA), after which coronal cells were manually removed with a finely drawn glass Pasteur pipette (Humagen Fertility Diagnostics, Charlottesville, VA, USA).The denuded oocytes were then assessed for nuclear status by an inverted microscope. Oocytes that released the first polar body were considered mature and used for ICSI.

### Oocyte morphology and intracytoplasmic sperm injection

For ICSI, oocytes were placed individually in 4 µL droplets of buffered medium (G-Mops™-V1, Vitrolife, Kungsbacka, Sweden). Sperm was placed in a central 4 µL droplet of polyvinylpyrrolidone solution (PVP, Irvine Scientific, Santa Ana, USA) in a 50×40 mm glass culture dish (WillCo-dish®, NJ, USA) covered with warm mineral oil (Ovoil™, Vitrolife, Kungsbacka, Sweden). Sperm injection was carried out on the heated stage (37ºC) of an inverted microscope (Eclipse TE 300; Nikon®, Tokyo, Japan) 40 hours after hCG triggering.

Immediately before sperm injection, we assessed oocyte morphology by an inverted microscope and recorded the following dysmorphisms: i. excessive cytoplasm granulation, ii. dark cytoplasm, iii. presence of vacuoles, iv. polar body fragmentation, v. perivitelline space dysmorphisms, vi. zona pellucida dysmorphisms, and vii. shape dysmorphisms. During the ICSI procedure, changes to membrane resistance to sperm injection were also recorded

### Statistical analysis

Continuous variables are given as means ± SEM, and proportions (%) are used for categorical variables. We compared proportions by the Chi-square or Fisher's exact test, when the expected frequency was five or less, and the results have been presented as proportions (%). To study the influence of the follicular diameter (large or small) on oocyte morphology, binary logistic regression models were conducted. The results are expressed as odds ratios (OR), 95% confidence intervals (CI) and p values. Results were considered significant at the 5% critical level (p<0.05). Data analysis was carried out using Minitab (version 14), a statistical analysis program.

### Ethical considerations

Written informed consent was obtained, in which patients agreed to share the outcomes of their cycles for research purposes. The study was approved by the Ethics Committee of the Federal University of Sao Paulo.

## Results

### Nuclear status and oocyte morphology

The overall numbers of aspirated follicles were 1016 and retrieved oocytes were 863, of which 604 were in the metaphase II (MII) stage, 95 in the metaphase I stage, 138 in prophase I stage and 26 were degenerated.

The diameters of the larger follicles ranged from 14 to 21 mm, and the diameters of the smaller follicles ranged from 11.6 to 14.6 mm. The mean diameter of the leading follicle group (19.1 ± 2.1) was significantly higher than the mean diameter of the smaller follicle group (13.0 ± 5.5, p<0.001).

A significantly higher percentage of oocytes derived from the leading follicle were in the MII-stage (100% vs. 70.0%, p<0.001); however, no significant differences were observed regarding the percentage of degenerated oocytes between the large (6.25%) and small follicle (5.0%, p=0.550) groups.

There were 11.5% of the small follicles that were in the metaphase I stage and 19.3% in the prophase I stage.

Regression analysis demonstrated a nearly twofold increase in the incidence of vacuoles in oocytes derived from the largest follicle of the cohort. We observed a trend toward a higher chance of presenting decreased membrane resistance to ICSI in oocytes derived from the leading follicle (Table 1).

There was no significant influence of the follicle diameter in the presence of excessive cytoplasm granulation, dark cytoplasm, perivitelline space dysmorphisms, polar body fragmentation, zona pellucida dysmorphisms, shape dysmorphisms or increased membrane resistance to ICSI (Table 1).

**Table 1:** Regression analysis of the influence of the follicular diameter on the incidence of oocyte defects


Oocyte defects	OR	CI lower	CI upper	P value

**Excessive cytoplasm granulation**	1.01	0.65	1.56	0.965
**Dark cytoplasm**	1.01	0.47	2.17	0.989
**Presence of vacuoles**	1.81	0.85	3.84	0.046
**Polar body fragmentation**	1.12	1.072	1.73	0.618
** Perivitelline space defects**	1.21	1.088	2.25	0.432
**Zona pellucida defects**	1.13	0.70	1.80	0.622
**Shape defects**	0.89	0.27	3.01	0.856
**Decreased membrane resistance to ICSI**	1.66	0.67	4.09	0.065
**Increased membrane**	0.73	0.38	1.41	0.346


OD;Odds ratio, CI; Confidence interval and OR; Refers to the larger follicle diameter.

### Fertilization and embryo quality

The fertilization rate (50.0% vs. 38.8%, p=0.038) and the percentage of top quality embryos (84.7% vs. 76.4%, p=0.040) were significantly higher for oocytes derived from the largest follicle of the cohort. However, the percentage of abnormal fertilized oocytes was equally distributed between the large follicle (15.0%) versus the small follicle (12.8%, p=0.550) groups.

When embryo selection was performed without taking into consideration the experimental group origin, we observed that embryos derived from the largest follicle (53.5%) were more commonly selected for transfer compared to the control group (33.8%, p<0.001).

## Discussion

The female gonad plays a key role in the differentiation and release of the mature oocyte for fertilization, embryo development and successful pregnancy. In monovular species, following the recruitment of a cohort of follicles, one follicle is selected for dominance and continues to grow while growth of the others is curtailed. Despite the critical importance of selection of the dominant follicle to ovarian function and fertility, why one follicle is selected from a group of similar follicles remains unknown (2).

In stimulated cycles, pharmacologic doses of gonadotropins create a supraphysiological hormonal environment that induces the growth of a cohort of follicles, which, under natural conditions, would become atretic and regress (2).

Here, we evaluated the developmental competence of oocytes retrieved from the lead follicle compared to those retrieved from smaller follicles of the cohort in COS cycles. The data showed that oocytes derived from the largest follicle presented a higher rate of both fertilization and top-quality embryos.

The most obvious sign that a follicle has been selected as dominant is a significant difference in size compared to the largest subordinate follicle (4). However, it has been previously suggested that selection of the dominant follicle is a progressive process and that the initial stages of selection occur before there is a perceptible difference in size (2).

A defining characteristic of the dominant follicle appears to be its greater capacity for estradiol production. Previous studies in domestic animals have shown that as soon as the dominant follicle is detected, it has higher concentrations of estradiol in the follicular fluid as soon as it becomes slightly larger than the largest subordinate follicle (4, 14). Previous studies have shown the presence of increased protease activity of insulin-like growth factor binding proteins (IGFBP) and increased concentration of free IGF-1 (15) in dominant follicles. In addition, it has been demonstrated that granulosa cells of the largest follicle acquire LH receptors shortly before the dominant follicle can be detected (10).

In the present study, despite the exposure to increased doses of exogenous gonadotropins, an increased percentage of immature oocytes were derived from smaller follicles rather than from larger follicles. It has been previously suggested that follicles containing immature oocytes after the administration of large doses of hCG must lack sufficient blood supply to receive the ovulatory stimulus or have insufficient LH receptors to induce oocyte maturation in vivo (16), which is substantiated by the frequent nonexpansion of the corresponding cumuli (17).

An increase in vascularity would give the follicle an advantage to receive a preferential supply of growth factors, gonadotropins, steroid precursors and other nutrients required for its continued development. The relationship between follicle vascularity and dominant follicle detection has been studied directly by Doppler ultrasonography in cattle (18, 19). Blood flow area begins to differentially increase in the future dominant versus subordinate follicle about one day before the beginning of diameter deviation. Differences in blood supply between follicles with different diameters could also explain the decreased rate of fertilization and top-quality embryos observed in our study for oocytes derived from smaller follicles. Rosen et al. (20) have previously demonstrated that the leading follicle was most likely to have a mature oocyte with increased fertilization and high quality embryo development capacity, however this study was performed in classic *in vitro* fertilization (IVF) cycles and the oocyte morphology could not be evaluated. In the present study we evaluated oocyte morphology immediately before ICSI; our data also suggest that although oocytes derived from larger follicles presented a higher developmental capacity, the incidence of vacuoles and decreased membrane resistance to ICSI was higher in oocytes derived from the lead follicle.

Oocyte quality has been regarded as a variable that influences the implantation potential of derived embryos (21-24). However, the predictive value of criteria used in these studies is still controversial. In fact, previous studies on the developmental outcome of oocytes with cytoplasmic abnormalities have suggested that cytoplasmic dysmorphisms are not related to fertilization or embryo quality (25, 26).

Vacuolization is probably the most apparent and dynamic cytoplasmic dysmorphism in human oocytes. Vacuoles vary in size as well as in number and, according to Van Blerkom et al. (27), they are membrane-bound cytoplasmic inclusions filled with fluid that is virtually identical with perivitelline fluid. It is assumed that vacuoles arise either spontaneously or by fusion of preexisting vesicles derived from the smooth endoplasmic reticulum and/or Golgi apparatus (28). Vacuoles appear to develop rapidly (within several minutes) around extrusions of the first polar body (23).

Interestingly, there is only one ICSI study (25) that found a impaired fertilization rate in vacuolized oocytes compared with vacuole-free MII gametes. All the other papers either published a normal fertilization rate (29) or did not deal with vacuolization as a separate e feature (26, 30).

According to Ebner et al. (23), during the ICSI, three distinct types of oolemma responses can be observed. Most of the injected oocytes show normal breakage of the membrane. A second type of response called 'difficult breakage' is characterized by delayed penetration. The third type of membrane response, sudden breakage of the oolemma without creation of a funnel may be observed. It has been described that the sudden breakage of the oolemma or decreased membrane resistance to ICSI is correlated with decreased rates of survival and fertilization (26, 29), however, once fertilization is achieved, apparently the oocyte development is normal

Conversely, our findings suggest that both vacuole formation and decreased membrane resistance to ICSI may occur in oocytes retrieved from over-aging follicles; however, fertilization and development of the embryo is not compromised by this feature.

The best way to evaluate oocyte quality is undoubtedly to evaluate the embryo implantation potential. In the present study however, neither the pregnancy nor the implantation rate could be compared between the groups, since in many cases embryos from both groups were transferred for the same patient. This is a limitation of the study, which could be avoided if exclusively elective single embryo transfers are performed. In our study we have used oocyte quality, fertilization capacity and embryo development as variables to evaluate oocyte competence.

## Conclusion

Together with previous reports our data suggest that intrafollicular mechanisms within the larger follicle of the cohort may allow it to amplify the responsiveness to the exogenous gonadotropins, leading to the formation of more competent oocytes with hiher fertilization and developmental capacities.
